# A Novel Requirement for Janus Kinases as Mediators of Drug Resistance Induced by Fibroblast Growth Factor-2 in Human Cancer Cells

**DOI:** 10.1371/journal.pone.0019861

**Published:** 2011-05-20

**Authors:** Catarina R. Carmo, Janet Lyons-Lewis, Michael J. Seckl, Ana P. Costa-Pereira

**Affiliations:** Imperial College London, Hammersmith Hospital Campus, London, United Kingdom; The University of Kansas Medical Center, United States of America

## Abstract

The development of resistance to chemotherapy is a major cause of cancer-related death. Elucidating the mechanisms of drug resistance should thus lead to novel therapeutic strategies. Fibroblast growth factor (FGF)-2 signaling induces the assembly of a multi-protein complex that provides tumor cells with the molecular machinery necessary for drug resistance. This complex, which involves protein kinase C (PKC) ε, v-raf murine sarcoma viral oncogene homolog B1 (B-RAF) and p70 S6 kinase β (S6K2), enhances the selective translation of anti-apoptotic proteins such as B-cell leukaemia/lymphoma-2 (BCL-2) and inhibitors of apoptosis protein (IAP) family members and these are able to protect multiple cancer cell types from chemotherapy-induced cell death. The Janus kinases (JAKs) are most noted for their critical roles in mediating cytokine signaling and immune responses. Here, we show that JAKs have novel functions that support their consideration as new targets in therapies aimed at reducing drug resistance. As an example, we show that the Janus kinase TYK2 is phosphorylated downstream of FGF-2 signaling and required for the full phosphorylation of extracellular signal-regulated kinase (ERK) 1/2. Moreover, TYK2 is necessary for the induction of key anti-apoptotic proteins, such as BCL-2 and myeloid cell leukemia sequence (MCL) 1, and for the promotion of cell survival upon FGF-2. Silencing JAK1, JAK2 or TYK2 using RNA interference (RNAi) inhibits FGF2-mediated proliferation and results in the sensitization of tumor cells to chemotherapy-induced killing. These effects are independent of activation of signal transducer and activator of transcription (STAT) 1, STAT3 and STAT5A/B, the normal targets of JAK signaling. Instead, TYK2 associates with the other kinases previously implicated in FGF-2-mediated drug resistance. In light of these findings we hypothesize that TYK2 and other JAKs are important modulators of FGF-2-driven cell survival and that inhibitors of these kinases will likely improve the effectiveness of other cancer therapies.

## Introduction

The development of drug resistance by tumor cells is responsible for the poor overall survival seen with most cancer types [1]. Amongst the plethora of drug resistance mechanisms lies fibroblast growth factor (FGF)-2 signaling. FGF-2 can provide cells with pro-survival and mitogenic signals, and confer broad-spectrum resistance to chemotherapeutic drugs [2], [3], [4]. De-regulated FGF-2 signaling has been associated with a variety of malignancies and elevated levels of this molecule in patient's serum were established as an independent poor prognostic factor for lymphoma, lung cancer and sarcoma patients [5], [6], [7]. In particular, FGF-2 was shown to promote drug resistance of small cell lung cancer (SCLC) cells through the formation of a multi-protein complex comprising protein kinase C (PKC) ε, v-raf murine sarcoma viral oncogene homolog B1 (B-RAF) and p70 S6 kinase β (S6K2). This depended on extracellular signal-regulated kinase (ERK) 1/2 activity [3], [8], [9]. The formation of this complex led to translational up-regulation of key anti-apoptotic proteins, which then conferred resistance to apoptosis and allowed cells to survive when challenged with cytotoxic drugs.

Most cytokines and many growth factors signal *via* Janus kinases (JAKs) and signal transducers and activators of transcription (STATs) [10], [11]. Constitutive STAT phosphorylation is characteristic of a wide variety of human cancer cell lines and primary tumors [12]. Overactivation of JAKs has also been implicated in tumorigenesis [13]. The fact that JAKs and STATs are activated by multiple mitogens has encouraged researchers to develop strategies to target them in cancer treatments. There is limited, somewhat confusing, evidence for JAK/STAT signaling downstream of FGF-2 and its relevance is unclear [14], [15], [16]. We and others [17] have observed de-regulated JAK/STAT expression in a variety of lung carcinoma and sarcoma cell lines. This and the fact that patients with elevated FGF-2 in the serum have poorer outcomes in the clinic led us to hypothesize that JAKs and/or STATs played a role in FGF-2 signaling-mediated drug resistance pathway(s). Here, we show that the Janus kinases JAK1, JAK2 and TYK2, but not their main downstream effectors STAT1, STAT3 or STAT5A/B, are required for FGF-2-mediated chemoprotection in U2OS osteosarcoma cells. This, in turn, opens new therapeutic avenues for cancers that are still difficult to control.

## Results

### FGF-2 protects osteosarcoma U2OS cells from cisplatin-mediated cell death through a mechanism that involves PKCε, B-RAF and S6K2 protein complexes

**Figure 1:**
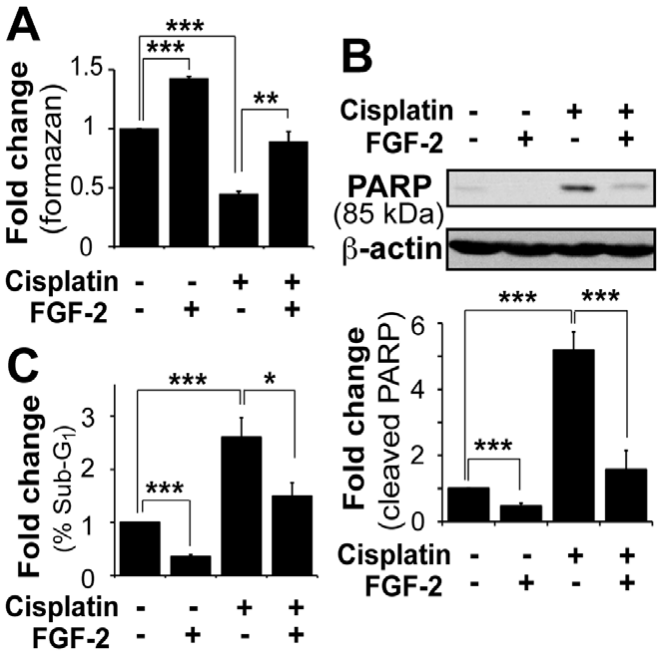
FGF-2 protected U2OS osteosarcoma cells from cisplatin-mediated cell killing. Cells were incubated in serum-free media overnight. After 4 h pre-treatment with FGF-2 (10 ng/ml), cells were treated with cisplatin (60 µM) overnight. **A.** Cell viability was measured using the WST-1 assay. Values correspond to tetrazolium salt WST-1 cleaved into colored formazan compounds by mitochondrial dehydrogenases. Mean±SEM of three independent experiments performed in triplicate are presented as fold induction relative to untreated controls. **B.** Proteins were separated on a 7.5% SDS-PAGE gel and western blotting analysis performed on total cell lysates (alive and dead cells) using antibodies against PARP. β-actin was used as a loading control. A representative western blot and mean±SEM of densitometric values (cleaved PARP) from three independent experiments are shown. **C.** Cells were permeabilized and the DNA stained with propidium iodide (PI). Cell cycle profiles were assessed by flow cytometry and the data mined using FlowJo. Mean±SEM of sub-G1 events from three independent experiments are expressed as fold-change of sub-G1 populations over the untreated control.

Since FGF-2 was shown to protect SCLC cells from cytotoxic drug-induced cell death [4], we initially sought to extend this observation to the distinct cancer cell type osteosarcoma U2OS cells. These were treated with the clinically relevant drug cisplatin in the presence and absence of FGF-2 (**Fig. 1**). The concentration of growth factor used was determined using ERK1/2 phosphorylation as a readout (**Fig. S1**). Cell death was assessed using the WST-1 cell viability assay, which yielded identical results to those obtained by cell counting with trypan blue (data not shown). Overnight treatment with 60 µM cisplatin killed 50% of the cells but pre-incubation with FGF-2 for 4 h prevented this, suggesting that FGF-2 protected U2OS cells against cisplatin (**Fig. 1A**). The WST-1 assay does not discriminate between apoptosis and necrosis. Moreover, the rescue from cisplatin could be attributed to proliferation, which was also induced by FGF-2, rather than purely to inhibition of cell death. We therefore monitored in parallel the induction of apoptosis using cleavage of caspase substrates such as poly(ADP-ribose) polymerase (PARP) and lamin B as read-outs (**Fig. 1B** and data not shown). In addition, cell cycle analysis by flow cytometry was used to quantify loss of DNA content, which can be used as another readout for apoptosis (**Fig. 1C**). Overnight treatment with cisplatin induced apoptosis in U2OS cells, which was manifested by the appearance of an 85 kDa PARP cleavage fragment (**Fig. 1B**) and an increase of the sub-G1 cell population (**Fig. 1C**). These events were prevented by pre-treating cells with FGF-2, which also reduced basal cell death detected in untreated samples. The graphs represent the average values obtained in three independent experiments. As observed for SCLC cells [8], the reduction of cell death by FGF-2 could be prevented by inhibition of mitogen-activated protein kinase kinase (MEK)/ERK activity with a selective inhibitor (**Fig. S2**), whilst inhibition of phosphatidylinositol 3-kinase (PI3K) and target of rapamycin complex (TORC) 1 signaling had no impact (data not shown). Thus FGF-2 exerted an anti-apoptotic effect in U2OS cells that was mediated by ERK1/2 and prevented the cytotoxic effects of cisplatin. Importantly, upon drug withdrawal, FGF-2-rescued cells remained viable in culture for several weeks (data not shown). This further supports the notion that FGF-2 can effectively promote the survival of cancer cells exposed to cytotoxic drugs.

**Figure 2:**
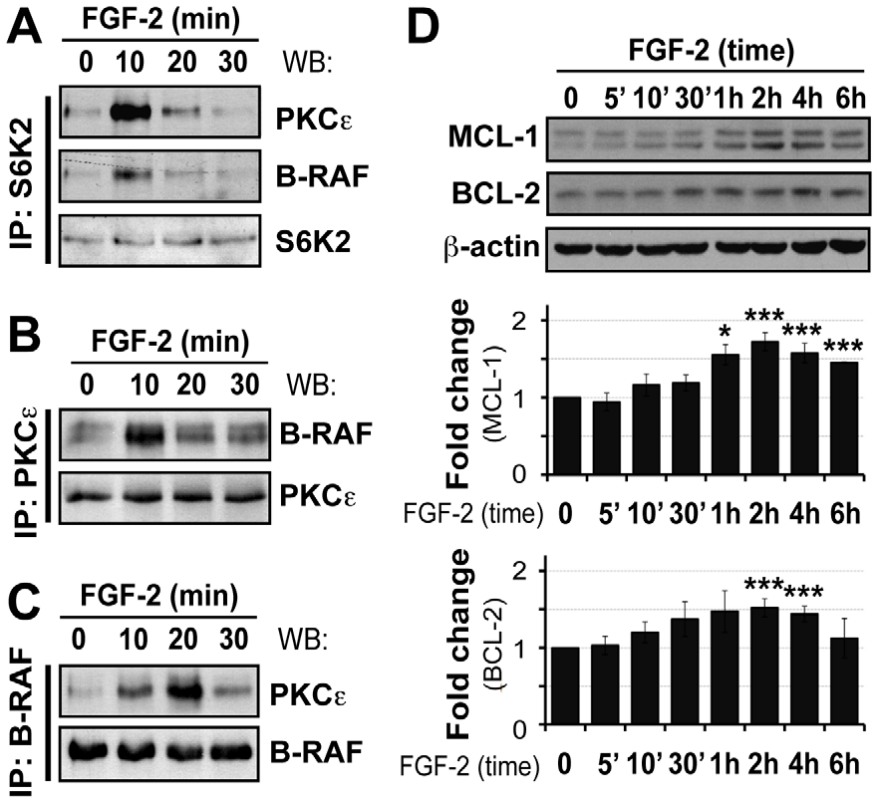
FGF-2 induced interactions between S6K2, PKCε and B-RAF, and upregulation of anti-apoptotic proteins in U2OS cells. Cells were serum-starved as before and after stimulation with FGF-2 (10 ng/ml), proteins extracted using the co-immunoprecipitation lysis buffer. Cell lysates were subjected to immunoprecipitation with (**A.**) S6K2, (**B.**) PKCε or (**C.**) B-RAF antibodies and proteins detected by western blotting as indicated. **D.** Western blot analysis was performed using total cell lysates and antibodies against MCL1 and BCL-2. β-actin was used as a loading control. Representative western blots and mean±SEM from three independent experiments are shown in graphs. ‘ – min.

In SCLC cells, FGF-2-mediated drug resistance pathways involves the formation of a protein complex involving PKCε, B-RAF and S6K2 [4]. We next asked whether FGF-2 could induce similar protein interactions in U2OS cells. Cells incubated in the presence or absence of FGF-2 for the times indicated were lyzed and S6K2 immunoprecipitated prior to western blotting for PKCε, B-RAF or S6K2 (**Fig. 2A**). B-RAF and PKCε associated with S6K2 in resting cells, an effect that was significantly increased by FGF-2 treatment for 10 min (**Fig. 2A**). The interactions were transient and no longer significant by 30 min post-FGF-2 treatment. To substantiate the notion that the three proteins interacted, we next immunoprecipitated either PKCε (**Fig. 2B**) or B-RAF (**Fig. 2C**) from cell lysates. We then performed western blotting to probe for these proteins and S6K2. While B-RAF and PKCε basal interaction increased 10 min after FGF-2 stimulation and subsequently persisted, we were unable to detect S6K2 in these immunoprecipitates (**Fig. 2B, 2C** and data not shown). This may be explained by lower expression levels of S6K2 compared to PKCε and B-RAF in U2OS cells, variation in stoichiometry of the associations or the formation of multiple, distinct, protein complexes. Overall the data suggest that FGF-2 promotes molecular interactions between PKCε, B-RAF and S6K2. In parallel with all immunoprecipitations, levels of the three proteins were also analyzed in whole cell extracts (**Fig. S3**). Interestingly, a small (1.5 to 1.7-fold) but highly reproducible and statistically significant increase of S6K2 was detected 10 min after the addition of FGF-2 whilst levels of PKCε, B-RAF, ERK1/2 and β-actin (the latter two used as loading controls) were unaffected (**Fig. S3** and also **S5**). Whether the increase in S6K2 signal is purely due to an increase in phosphorylation or stabilization of the protein remains to be determined. Phosphorylation of ERK1/2 was also monitored to ensure the functionality of FGF-2 (**Fig. S3**). To further verify that U2OS cells behaved like SCLC in response to FGF-2 pro-survival signaling we also determined if an increase in anti-apoptotic proteins could be detected [8]. As predicted, FGF-2 increased B-cell leukaemia/lymphoma-2 (BCL-2), BCL-2-like 1 protein (BCL-x_L_) and X-linked inhibitor of apoptosis protein (XIAP) expression levels but, in addition, we also observed induction of another important anti-apoptotic protein, namely myeloid cell leukemia sequence (MCL) 1, in U2OS cells (**Fig. 2D** and data not shown).

### Downregulation of JAK1, JAK2 or TYK2, but not STAT1, STAT3 or STAT5, inhibits FGF-2-mediated drug resistance in U2OS cells

**Figure 3:**
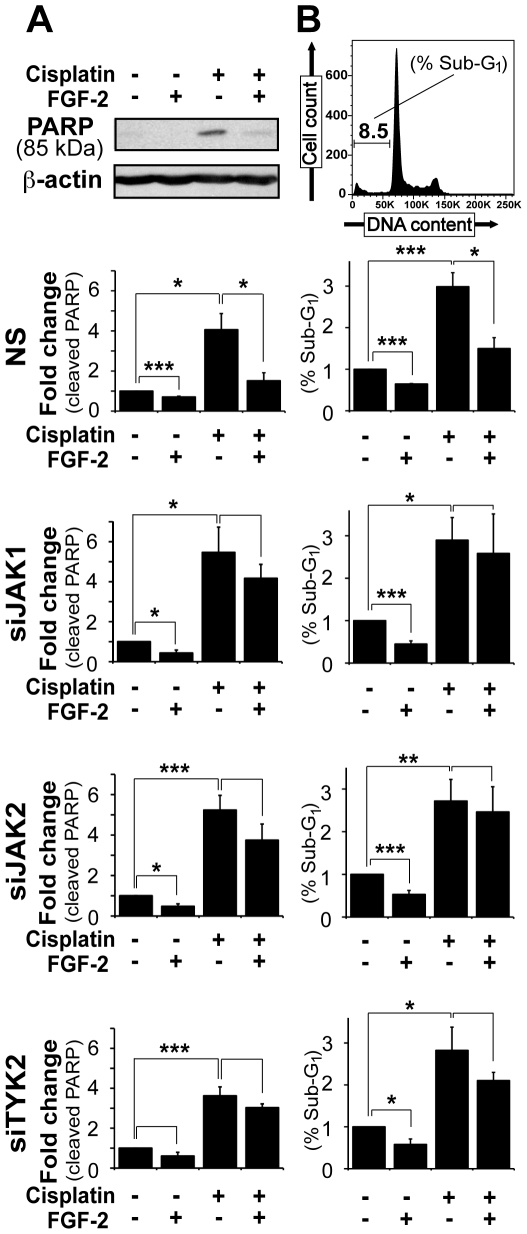
Knockdown of JAK1, JAK2 or TYK2 blocked the ability of FGF-2 to prevent cisplatin-induced apoptosis in U2OS cells. Cells were transfected with 75 nM of siRNA against JAK1, JAK2 or TYK2. Untransfected cells and cells transfected with non-specific siRNA (NS) were used as controls. After 48 h, cells were re-plated (2×10^5^ cells/well) and incubated in serum-free media overnight. After 4 h pre-treatment with FGF-2 (10 ng/ml), cells were treated with cisplatin (60 µM) overnight. **A.** Mean±SEM of densitometric values (cleaved PARP) from three experiments are shown. **B.** Cell cycle profiles of three independent experiments are graphically represented as mean±SEM of sub-G1 populations. The top panels exemplify the data and correspond to control cells.

**Figure 4:**
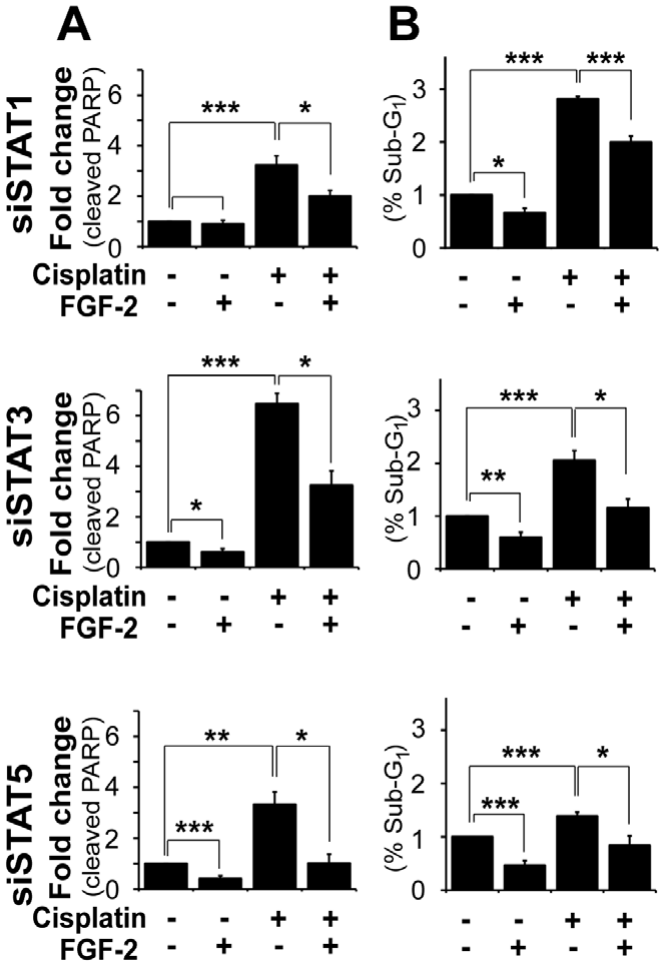
Silencing STAT1, STAT3 or STAT5A/B did not impair the ability of FGF-2 to prevent cisplatin-induced apoptosis in U2OS cells. Cells were transfected with 75 nM of siRNA against STAT1, STAT3 or STAT5A/B. Untransfected cells and cells transfected with non-specific siRNA (NS) were used as controls (c.f. **Fig. 3**). Cells were treated as in Figure 3. **A.** Mean±SEM of densitometric values (cleaved PARP) from three experiments are shown. **B.** Cell cycle profiles of three independent experiments are graphically represented as mean±SEM of sub-G1 populations.

Many cancer cell lines used in research have de-regulated JAK/STAT signaling. This reflects the *in vivo* situation as the same is observed in human tumor specimens. Despite their traditional roles in immune responses JAKs and STATs are being increasingly recognized as important molecules in cancer biology. Our preceding data demonstrated that FGF-2 induced interactions between B-RAF, PKCε and S6K2, increased the expression of key anti-apoptotic molecules and protected U2OS cells from cisplatin-induced apoptosis. We next asked whether ubiquitously expressed JAK family members and/or their substrate STATs were part of this signaling pathway. To address this question, RNA interference (RNAi) was induced using short interfering (si) oligonucleotides against JAK1, JAK2, TYK2, STAT1, STAT3 and STAT5A/B. Throughout this study we have used siRNA pools each comprising four siRNA oligonucleotides directed against different regions of the same target molecules. All siRNA pools were validated by deconvolution and western blotting using antibodies raised against the known target as well as closely related molecules, as previously described in our laboratory [18]. Silencing efficiency and specificity of pooled siRNAs are shown in **Figure S4**. U2OS cells transfected with siRNA were treated with or without FGF-2 for 4 h and subsequently exposed to cisplatin (**Fig. 3** and **4**). Approximately 50% of U2OS cells that had silenced JAK1 (siJAK), JAK2 (siJAK2) or TYK2 (siTYK2) treated with cisplatin underwent apoptosis and this could not be prevented by pre-treating these cells with FGF-2 (**Fig. 3**). Apoptosis was assessed using the WST-1 assay (data not shown), or using PARP cleavage (**Fig. 3A**) and DNA fragmentation (**Fig. 3B**) as readouts. In stark contrast, the increased PARP cleavage and sub-G1 cell population induced by cisplatin was prevented by FGF-2 in control cells transfected with a non-specific siRNA (NS) and in untransfected cells. Thus, each of the analyzed JAKs was required for efficient FGF-2-mediated chemoprotection from cisplatin-induced apoptosis.

We next examined whether JAKs might act through their canonical downstream targets STAT1, STAT3 or STAT5. However, silencing each of these molecules individually had no effect on FGF-2-induced drug resistance. Indeed, PARP cleavage (**Fig. 4A**) and sub-G1 events (**Fig. 4B**) triggered by cisplatin remained significantly reduced in cells pretreated with FGF-2 regardless of the levels of STAT1, STAT3 or STAT5A/B.

### FGF-2 induces TYK2 but not STAT phosphorylation

**Figure 5:**
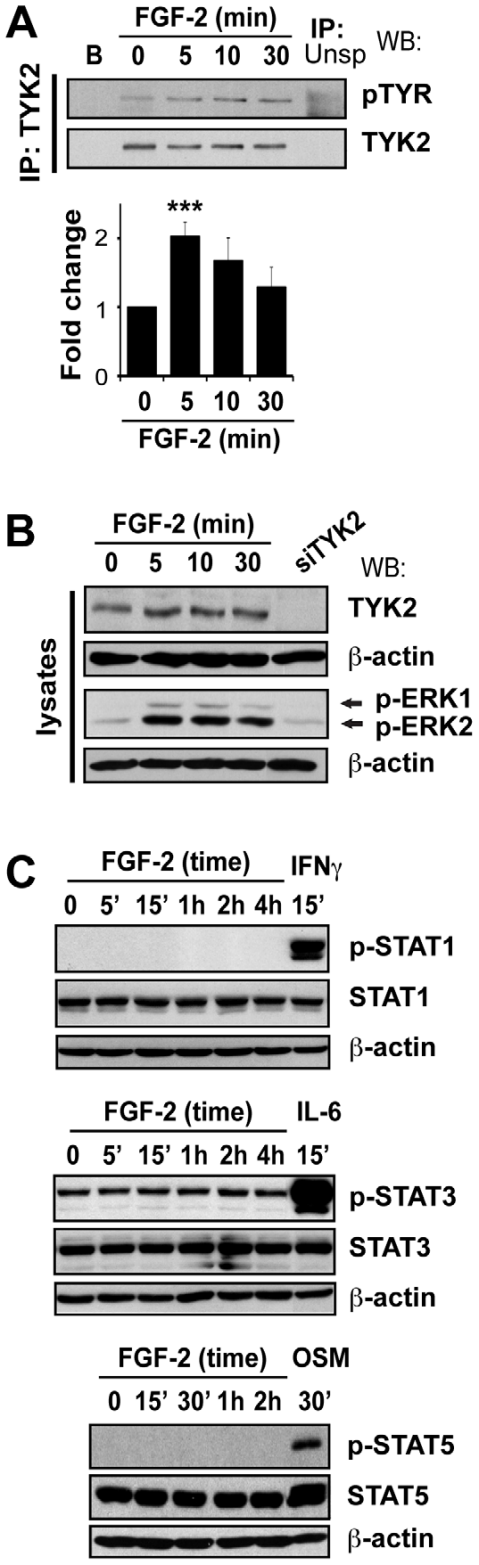
FGF-2 treatment leads to phosphorylation of TYK2, but not STAT1, STAT3 or STAT5A/B. **A.** FGF-2 did not induce STAT1, STAT3, or STAT5 phosphorylation. U2OS cells were serum-starved overnight and then stimulated with FGF-2 for the indicated times. Cells treated with IFN-γ (500 IU/ml), IL-6 (200 ng/ml)/sIL-6R (250 ng/ml) and OSM (50 ng/ml) were used as positive controls for activation of STAT1, STAT3 and STAT5, respectively. Proteins were analyzed as before using antibodies against phosphorylated STAT1 (Tyr^701^), phosphorylated STAT3 (Tyr^705^), phosphorylated STAT5 (Tyr^694^) and antibodies that recognize both phosphorylated and unphosphorylated proteins. **B.** FGF-2 induced the phosphorylation of TYK2 in U2OS cells. Cells were incubated in serum-free media and then treated with FGF-2 (10 ng/ml) for the indicated times. TYK2 was immunoprecipitated and western blotting analysis was performed using antibodies against phosphorylated tyrosines and total TYK2. TYK2 was used as a loading control. **C.** Total cell lysates used to immunoprecipitate TYK2 and from cells transfected with siTYK2 were separated on a 7.5% SDS-PAGE gel and analyzed by western blot. Membranes were probed for TYK2, pERK1/2-Thr^202/185^/Tyr^204/187^ and total ERK1/2. β-actin was used as a loading control. ‘ – min.

STAT1, STAT3 and STAT5A/B are natural substrates of the JAKs, but our preceding data indicate that they may not be required for FGF-2-induced pro-survival signaling. If this were indeed true, then one would expect FGF-2 to be unable to activate STATs, a process that involves tyrosine phosphorylation on specific residues for which phosphospecific antibodies are available [19]. Consequently, we next investigated whether STATs were modified downstream of FGF-2 by analyzing tyrosine phosphorylation of STAT1 (Tyr^701^), STAT3 (Tyr^705^) and STAT5A/B (Tyr^694^) by western blotting. In agreement with our previous findings (**Fig. 4**), FGF-2 failed to induce activation of STAT1, STAT3 or STAT5 in U2OS cells (**Fig. 5A**). Nevertheless, as expected, these proteins could be phosphorylated by Interferon (IFN)- γ, interleukin (IL)-6, or oncostatin M (OSM), which were used as positive controls (**Fig. 5A**).

A potential explanation of these findings includes the possibility that FGF-2 simply fails to activate the JAKs, a process that also involves tyrosine phosphorylation. To determine whether JAKs might be activated by FGF-2, we decided to focus our attention on TYK2, one of the lesser-studied JAKs that is widely expressed in many cell types. U2OS cells were treated with FGF-2 for different periods of time and TYK2 was immunoprecipitated. TYK2 tyrosine phosphorylation was determined using antibodies against phosphorylated tyrosines (4G10 and PY20, 1∶1 mix) and TYK2 protein expression with an anti-TYK2 antibody. FGF-2 induced a transient 2-fold increase in TYK2 tyrosine phosphorylation, which peaked at 5 min and returned close to basal levels by 30 min (**Fig. 5B**). No phospho-TYK2 or TYK2 was seen in control samples (immunoprecipitations with beads only, or with an irrelevant antibody). In parallel, whole cell lysates used for the immunoprecipitation and lysates from siTYK2-tranfected cells (antibody control) were analyzed (**Fig. 5C**). The data thus suggest that TYK2 is phosphorylated, and potentially activated, downstream of FGF-2. These results together with those presented in **Figure 3** are entirely consistent with a role for TYK2 downstream of FGF-2 that does not depend on the simultaneous phosphorylation/activation of STAT1, STAT3 and STAT5. It remains to be established if JAK1 and JAK2 are also phosphorylated downstream of FGF-2.

### TYK2 binds to PKCε and B-RAF upon FGF-2 treatment and is required for full phosphorylation of ERK1/2 and MCL1 induction

**Figure 6:**
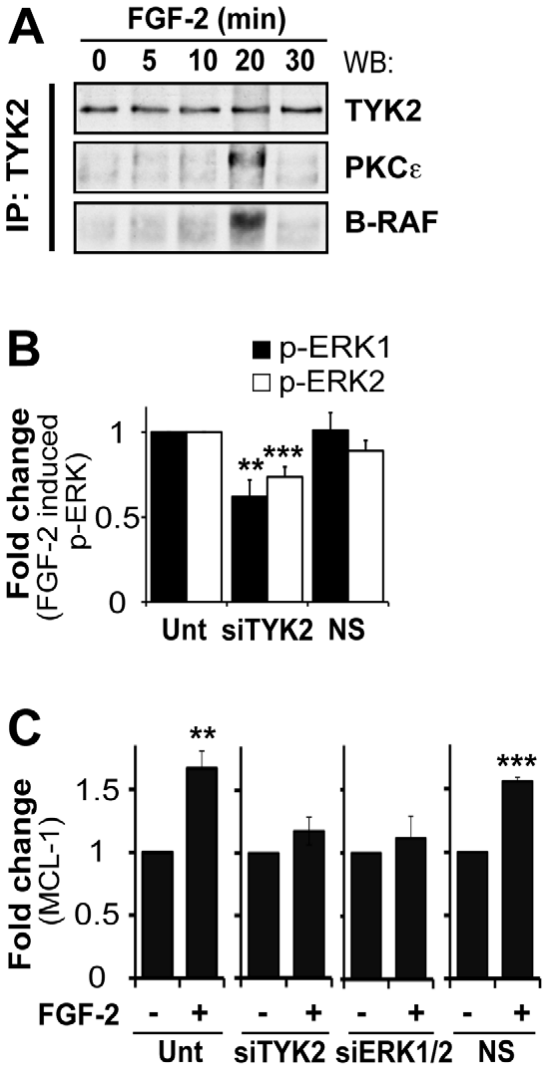
TYK2 played an important role in FGF-2-mediated drug resistance. **A.** FGF-2 induced interaction of TYK2 with PKCε and B-RAF in U2OS cells. Cells were treated as previously described and proteins isolated using co-immunoprecipitation buffer. TYK2 was immunoprecipitated from whole cell lysates and immunoprecipitates were analyzed using western blots. Membranes were probed for PKCε, B-RAF and TYK2, the latter used here as a loading control. **B.** and **C.** TYK2 silencing in U2OS cells led to decreased ERK1/2 phosphorylation and inhibited the upregulation of MCL1 in response to FGF-2. Cells were transfected with siTYK2 as before. Untransfected cells and cells transfected with a non-specific siRNA (NS) were used as controls. After 48 h, cells were treated as described previously and then stimulated with FGF-2 for (**B**) 10 min and (**C**) 4 h. **B.** Proteins were analyzed using antibodies against p-ERK1/2 (Thr^202/185^/Tyr^204/187^) and total ERK1/2. Values correspond to phospho-ERK1 and -ERK2 levels. **C.** Western blotting analysis was performed using total cell lysates and anti-MCL1 antibody. β-actin was used as a loading control and for normalization. Mean±SEM of densitometric values of three independent experiments are shown.

The preceding data prompted us to determine if TYK2 interacted with B-RAF, PKCε and S6K2. It was not trivial to detect TYK2 in S6K2, B-RAF and PKCε protein complex(es) presumably due to low expression levels for this protein in U2OS cells. TYK2 was therefore immunoprecipitated after treatment with FGF-2 and the immunoprecipitates analyzed by western blotting with antibodies against B-RAF, PKCε and S6K2 instead. Upon FGF-2 stimulation TYK2 briefly associated with B-RAF and PKCε (**Fig. 6A**). The transient nature of these associations may also explain why it was also difficult to detect S6K2 in these immunocomplexes. The control lysates are shown in **Figure S5**.

FGF-2-mediated chemoprotection required ERK1/2 activity (**Fig. S2**). To ascertain if this was dependent on TYK2, U2OS cells were transfected with siTYK2, subsequently treated with FGF-2 and ERK1/2 phosphorylation assessed by western blot (**Fig. 6B**). When compared to untransfected cells, RNAi against TYK2 significantly reduced ERK1/2 phosphorylation (by 40% for ERK1 and 25% for ERK2), a phenomenon not observed in cells transfected with non-specific siRNA. This prompted us to investigate next whether the upregulation of anti-apoptotic proteins by FGF-2 was impaired upon TYK2 silencing. RNAi was performed as before and, as an example, the expression of MCL1 monitored by western blot (**Fig. 6C**). Untransfected cells and cells transfected with either a non-specific siRNA or siERK1/2 were used as controls. In untransfected cells, or cells transfected with a non-specific control, FGF-2 lead to a significant increase in MCL1 protein levels. As predicted, siRNA against ERK1/2 inhibited the upregulation of MCL1 protein. Importantly, MCL1 expression did not increase in response to FGF-2 in cells with decreased levels of TYK2, suggesting that this JAK is required for the upregulation of MCL1.

## Discussion

There are many molecular mechanisms by which cells can become resistant to cytotoxic drugs and thus different types of drug resistance need to be tackled to subdue cancer cells. FGF-2 has been identified as an adverse prognostic factor for many cancer patients [5], [6], [7] and Seckl and Pardo have demonstrated its relevance for drug resistance [3], [4], [8], [9], [20].

The output of JAK/STAT signaling pathways impacts far beyond the cytokine realm. It is widely accepted that JAK/STAT signaling is highly context-dependent [18], [21], [22], [23], [24], that it is modular in nature and that it involves both cross-talk and cross-recruitment of other pathways [11], [25]. STATs are not usually found mutated but are frequently seen constitutively activated in cancer cells [12], [19]. In contrast, JAKs are often mutated and thus de-regulated, particularly in hematological malignancies [26], [27]. To our knowledge, JAKs have never been directly implicated in drug resistance. It is, however, widely recognized that many cancers are accompanied by inflammation, which is mediated by cytokines and, consequently, JAK and STAT activity [28]. De-regulated inflammation, in turn, has been implicated in many aspects of cancer development and progression, including drug resistance [12]. Interestingly, JAKs can have contradictory cellular responses and, as mentioned above, this depends on the trigger and on the cellular context [13], [29]. Proliferative responses can be directly mediated by the STATs, or by other molecules such as ras viral oncogene homolog (RAS), ERKs, v-src sarcoma viral oncogene homolog (SRC) and PI3K [reviewed in [30]]. The regulation of cytokine responses by the JAKs is STAT-dependent but JAK-dependent STAT-independent cellular responses have also been reported. Indeed, JAK1 and JAK2 modulate expression of BCL-2 family members, molecules crucial for cellular life and death decisions, independently of STAT activity [31], [32]. Studies using TYK2-deficient cell lines suggested that TYK2 was critical for the responses to IFN-α/β and some cytokines. In contrast, knockout mice, despite being more susceptible to viral infections, had their cytokine responses largely intact [33]. Studies on a patient with a naturally occurring TYK2 deficiency, however, suggested that TYK2 was required for full cytokine signaling [reviewed in [13]]. The precise roles of TYK2, even within cytokine responses, remain thus to be fully elucidated.

Here, we have shown that in U2OS cells TYK2 is rapidly phosphorylated downstream of FGF-2 (**Fig. 5B**), is required for full activation of ERK1/2 (**Fig. 6B**) and for the induction of MCL1 (**Fig. 6C**). Moreover, TYK2, JAK1 and JAK2 are required for FGF-2-mediated protection against cisplatin (**Fig. 1** and **3**). This effect appears to be independent of the STATs, as silencing of STAT1, STAT3 or STAT5A/B did not block the survival effects mediated by FGF-2 (**Fig. 1** and **4**). Each STAT was silenced independently and it is possible that concomitant knockdown of all three STATs, or indeed of other STAT family members, may impact on FGF-2 drug resistance. The fact that no phosphorylation of STATs can be detected following FGF-2 treatment suggests, however, otherwise (**Fig. 5A**). It is still not known how TYK2 is phosphorylated downstream of FGF-2. TYK2 may be directly recruited to the FGF receptor, or it may be phosphorylated further downstream by another kinase such as SRC [30]. The lack of STAT phosphorylation downstream of FGF-2 and lack of impact on FGF-2 drug resistance (**Fig. 4** and **5A**) also suggests that cytokines are not involved in drug resistance pathways triggered by this growth factor. The role of TYK2 in FGF-2-mediated drug resistance may be enzymatic and/or structural (**Fig. 5B** and **6A**). Indeed a structural role for TYK2 has been attributed to TYK2 in IFN-α/β responses where its presence was not only required for signaling but also for the cell surface expression of the IFNAR1 chain [34]. Further work is, therefore, necessary to determine whether TYK2 kinase activity and/or structure is required to mediate the FGF-2-induced pro-survival and drug resistance phenotype.

As for SCLC cells [4], treatment of U2OS cells with FGF-2 induces formation of protein complexes involving B-RAF, PKCε and S6K2 (**Fig. 2A–2C**). Conceptually, FGF-2 can lead to the formation of three different dimers (B-RAF/PKCε; B-RAF/S6K2; S6K2/PKCε) and/or the formation of a trimer (B-RAF/PKCε/S6K2). The precise stoichiometry of the protein complexes induced by FGF-2 remains to be determined. The interaction between these three proteins occurs within 10 min of FGF-2 treatment and is transient in nature, decreasing significantly by 30 min. Although the precise role of the complex is still being unraveled, it is tempting to speculate that its function may be to provide a signaling platform where these kinases can assemble and undergo specific phosphorylation events required for further downstream biological responses that eventually lead to cell survival. Interestingly, S6K2 levels quickly increase after FGF-2 treatment indicating that, in addition to inducing S6K2 phosphorylation, FGF-2 may also stabilize S6K2 (**Fig. S3** and **S5**). To the best of our knowledge, this is a new finding that warrants a more detailed investigation. TYK2 immunoprecipitation following FGF-2 treatment co-immunoprecipitates PKCε and B-RAF, further suggesting an important role for TYK2 in FGF-2-mediated drug resistance (**Fig. 6A**). Accordingly, silencing either TYK2, JAK1 or JAK2 using RNAi blocked FGF-2 drug resistance against cisplatin (**Fig. 3**). It will be particularly pertinent to determine if the formation of protein complexes involving PKCε, B-RAF and S6K2 depends on TYK2 since TYK2 phosphorylation is relatively quick and can be detected 5 min post-FGF-2 treatment. In addition, we have identified MCL1 as a TYK2-regulated molecule. Indeed, under these experimental conditions, increased expression of MCL1 following FGF-2 depended on intact TYK2 (**Fig. 6C**). This is likely an important finding as, like BCL-2, MCL1 does not induce proliferation but rather promotes cancer cell viability by blocking apoptosis [35]. In addition, it is one of the genes highlighted in a study by Meyerson and co-workers that was designed to identify genomic areas frequently altered in human cancers [36].

Collectively, our data suggest that TYK2, JAK1 and JAK2 are novel targets that may prove useful to circumvent drug resistance mediated by FGF-2, a growth factor often secreted by cancer cells, or their supporting tissues, and elevated in cancer patients. The precise molecular mechanisms involved are thus currently under investigation in our laboratory.

## Materials and Methods

### Cell culture

U2OS cells from the ATCC were grown in Dulbecco's Modified Eagle's Medium (DMEM) supplemented with 10% (v/v) fetal calf serum (FCS) (FirstLink), 2 mM L-glutamine, 50 units/ml penicillin and 50 µg/ml streptomycin (Biowhittaker) in a humidified atmosphere of 10% CO_2_ at 37°C, as previously described [18]. FGF-2 was from Calbiochem.

### Antibodies

Anti-TYK2 (C-20), STAT3 (N-terminal), STAT5A (L-20), STAT5B (G-2), ERK1 (subdomain XI), B-RAF (H145) and BCL-2 (100) were purchased from Santa Cruz Biotechnology. STAT1 (N-terminal) and PY20 antibodies were obtained from BD Transduction Laboratories. Antibodies against phosphorylated STAT1 (pSTAT1-Tyr^701^), phosphorylated STAT3 (pSTAT3-Tyr^705^), phosphorylated STAT5 (pSTAT5-Tyr^694^), phosphorylated ERK1/2 (pERK1/2-Thr^202/185^/Tyr^204/187^) and PARP were bought from Cell Signaling Technology. Anti-MCL1 was from BD Pharmingen. 4G10 and PKCε antibodies were from Millipore. The antibody against β-actin was obtained from Sigma-Aldrich. Anti-S6K2 (430-C-terminus) used for immunoprecipitations was from Bethyl and the one used for blotting was a gift from Dr I. Gout (University College London, London, UK).

### Protein analysis

Proteins were isolated using Schindler lysis buffer [18] and concentrations determined using a BioRad assay according to the manufacturer's instructions.

### Immunoprecipitation

Cells were serum starved overnight [DMEM/0.5% (v/v) FCS], incubated in DMEM for 2 h and stimulated with FGF-2 (10 ng/ml) for 5–30 min. After extracting proteins with Schindler's lysis buffer, 3 mg were incubated rotating at 4°C for 1 h with 1.6 µg/ml anti-TYK2, followed by 1 h with protein A-agarose beads. Immunoprecipitates were washed extensively with lysis buffer and boiled before separation on a 7.5% SDS-PAGE gel. Co-immunoprecipitations were done as described previously using 0.3 mg of protein [4]. Importantly, prior to immunoprecipitation experiments all antibodies were tested for specificity using RNAi and all experiments have included controls for non-specific protein binding to the beads. For clarity and conciseness purposes, gels have been cropped and/or presented in a graphic format. This has in no way compromised the integrity of the results and all gels are available for scrutiny.

### Drug resistance assays

Cells were starved overnight and then incubated with or without FGF-2 (10 ng/ml) for 4 h prior to addition of cisplatin. When carrying out the assay after inducing RNAi, cells were counted and re-plated. Cell death was measured using WST-1 (Calbiochem), as directed by the manufacturer. Apoptosis was also assessed by western blotting and by flow cytometry using PARP cleavage and DNA fragmentation as readouts, respectively. For the latter, cells were incubated with propidium iodide (50 µg/ml) and RNase A (10 µg/ml), cell cycle profiles analyzed and sub-G1 peaks corresponding to apoptotic cells quantified using FlowJo (Tree Star, Inc., USA).

### RNA interference (RNAi)

This was done as previously described [18].

### Statistical analysis

Mean±standard error (SEM) of three independent experiments were calculated. Student's t-test was used to determine the statistical significance of the differences observed between conditions. A two-tailed *p*-value below 0.05 was considered significant. * – p<0.05, ** - p<0.01, *** – p<0.005.

## Supporting Information

Figure S1 **FGF-2 induced ERK1/2 phosphorylation in U2OS cells in a dose- and time-dependent manner.** U2OS cells were serum-starved (0.5% FCS) overnight and then incubated in serum-free media for 1 hour. Cells were stimulated with FGF-2 for 10 minutes with (**A.**) the indicated concentrations or with (**B.**) 10 ng/ml of FGF-2 for the indicated times. Proteins were extracted and separated on a 10% SDS-PAGE gel. Western blotting analysis of total cell lysates was performed using antibodies against pERK1/2-Thr^202/185^/Tyr^204/187^ and total ERK1/2. β-actin was used as a loading control. Representative western blots and mean±SEM of densitometric values of three independent experiments are shown in graphs. Values are expressed as fold change relative to untreated controls. Statistical analysis was performed using Student's t-test (* – p<0.05, ** – p<0.01, *** – p<0.005 *versus* untreated control). ‘ – min.
(TIF)

Figure S2 **FGF-2-mediated drug resistance requires ERK1/2 phosphorylation.** Cells were serum-starved (0.5% FCS) overnight, incubated in serum-free media for 1 hour and then pre-treated with 0.5 µM of PD0325901 or with drug vehicle [0.005% (v/v) DMSO] for 30 minutes. **A.** Cells were stimulated with 10 ng/ml of FGF-2 for 10 minutes. Proteins were extracted and separated on a 10% SDS-PAGE gel. Western blotting analysis was performed on total cell lysates using antibodies against pERK1/2-Thr^202/185^/Tyr^204/187^ and total ERK1/2. β-actin was used as a loading control. **B.** After 4 hours pre-treatment with FGF-2, cells were treated with cisplatin (60 µM) overnight, proteins were extracted from alive and dead cells, run on a 7.5% SDS-PAGE gel and analyzed by western blotting using a PARP antibody that recognizes cleaved PARP. **C.** Cells were treated as in (B.). After 18 h of cisplatin treatment, cells were harvested, permeabilized and the DNA stained. Apoptosis was assessed by flow cytometry using loss of DNA content as readout (% sub-G1 population). Mean±SEM from three independent experiments are shown in the graphs. Values are expressed as fold change over untreated controls (without PD0325901 and FGF-2). Statistical analysis was performed with Student's t-test (* – p<0.05, ** – p<0.01, *** – p<0.005 *versus* untreated control). [Grey asterisks in (A.): compare the indicated samples].
(TIF)

Figure S3 **FGF-2 induced interactions between S6K2, PKCε and B-RAF.** Whole cell lysates used to immunoprecipitate S6K2, PKCε and B-RAF were separated on a 7.5% SDS-PAGE gel and analyzed by western blot. Membranes were probed for PKCε, B-RAF, S6K2, pERK1/2-Thr^202/185^/Tyr^204/187^ and total ERK1/2. β-actin was used as a loading control. Representative western blots and mean±SEM of densitometric values from three independent experiments are shown in the graph. Values are expressed as fold change over untreated controls. Statistical analysis was performed with Student's t-test (* – p<0.05, *** – p<0.005 *versus* untreated controls).
(TIF)

Figure S4 **siRNA oligonucleotides can specifically knockdown TYK2, STAT1, STAT3 and STAT5A/B in U2OS cells.** U2OS cells were transfected with 75 nM siRNA using DharmaFECT II. After 48 hours, proteins were extracted and separated on a 7.5% SDS-PAGE gel. Total cell lysates were western blotted as indicated. Cells were transfected with (**A.**) siTYK2, or (**B.**) with siRNA molecules targeting STAT1, STAT3 or STAT5A/B. Untransfected cells (Unt), or cells transfected with a non-specific siRNA (NS) were used as controls. Mean±SEM of densitometric values of three independent experiments are shown in graphs. Values are expressed as fold change relative to untransfected controls. Statistical analysis was performed with Student's t-test (*** – p<0.005 *versus* untransfected control). Unt – untransfected, NS – non-specific siRNA, T2 – TYK2, ST1 – STAT1, ST3 – STAT3, ST5 – STAT5A/B.
(TIF)

Figure S5 **FGF-2 induced interactions between TYK2, PKCε and B-RAF.** Whole cell lysates used to immunoprecipitate TYK2 were separated on a 7.5% SDS-PAGE gel and analyzed on a western blot. Membranes were probed for PKCε, B-RAF, S6K2 and TYK2. β-actin was used as a loading control. Representative western blots and mean±SEM of densitometric values from three independent experiments are shown in the graph. Values are expressed as fold change over untreated controls. Statistical analysis was performed with Student's t-test (*** – p<0.005 *versus* untreated controls).
(TIF)
