# Supplementary figures and images for: A Novel Requirement for Janus Kinases as Mediators of Drug Resistance Induced by Fibroblast Growth Factor-2 in Human Cancer Cells
Source: PLoS One. 2011 May 20;6(5):e19861. doi: 10.1371/journal.pone.0019861 (PMC3098828; doi:10.1371/journal.pone.0019861)

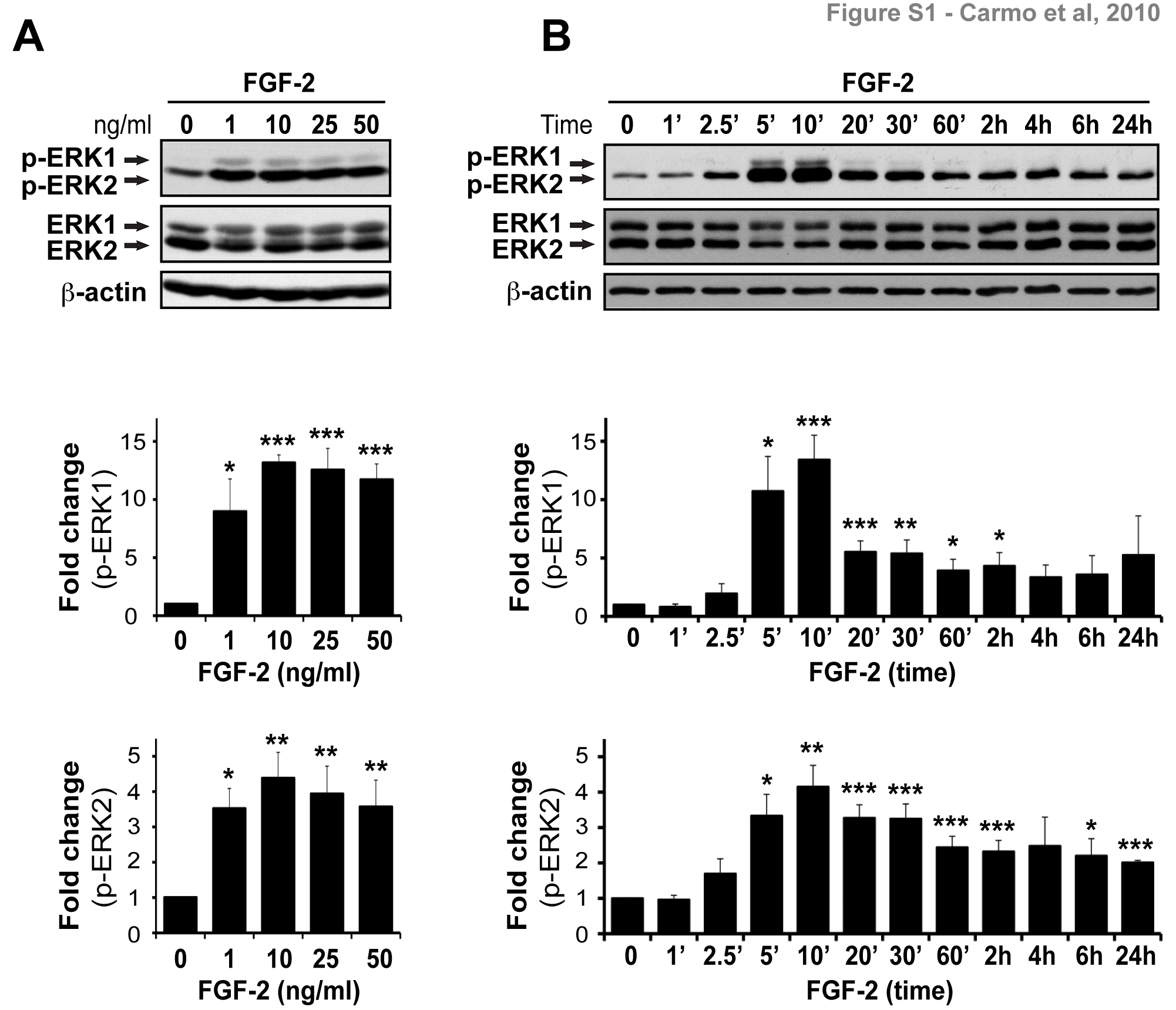

Supplement: Figure S1 — FGF-2 induced ERK1/2 phosphorylation in U2OS cells in a dose- and time-dependent manner. U2OS cells were serum-starved (0.5% FCS) overnight and then incubated in serum-free media for 1 hour. Cells were stimulated with FGF-2 for 10 minutes with (A.) the indicated concentrations or with (B.) 10 ng/ml of FGF-2 for the indicated times. Proteins were extracted and separated on a 10% SDS-PAGE gel. Western blotting analysis of total cell lysates was performed using antibodies against pERK1/2-Thr202/185/Tyr204/187 and total ERK1/2. β-actin was used as a loading control. Representative western blots and mean±SEM of densitometric values of three independent experiments are shown in graphs. Values are expressed as fold change relative to untreated controls. Statistical analysis was performed using Student's t-test (* – p<0.05, ** – p<0.01, *** – p<0.005 versus untreated control). ‘ – min. (TIF) [file pone.0019861.s001.tif]

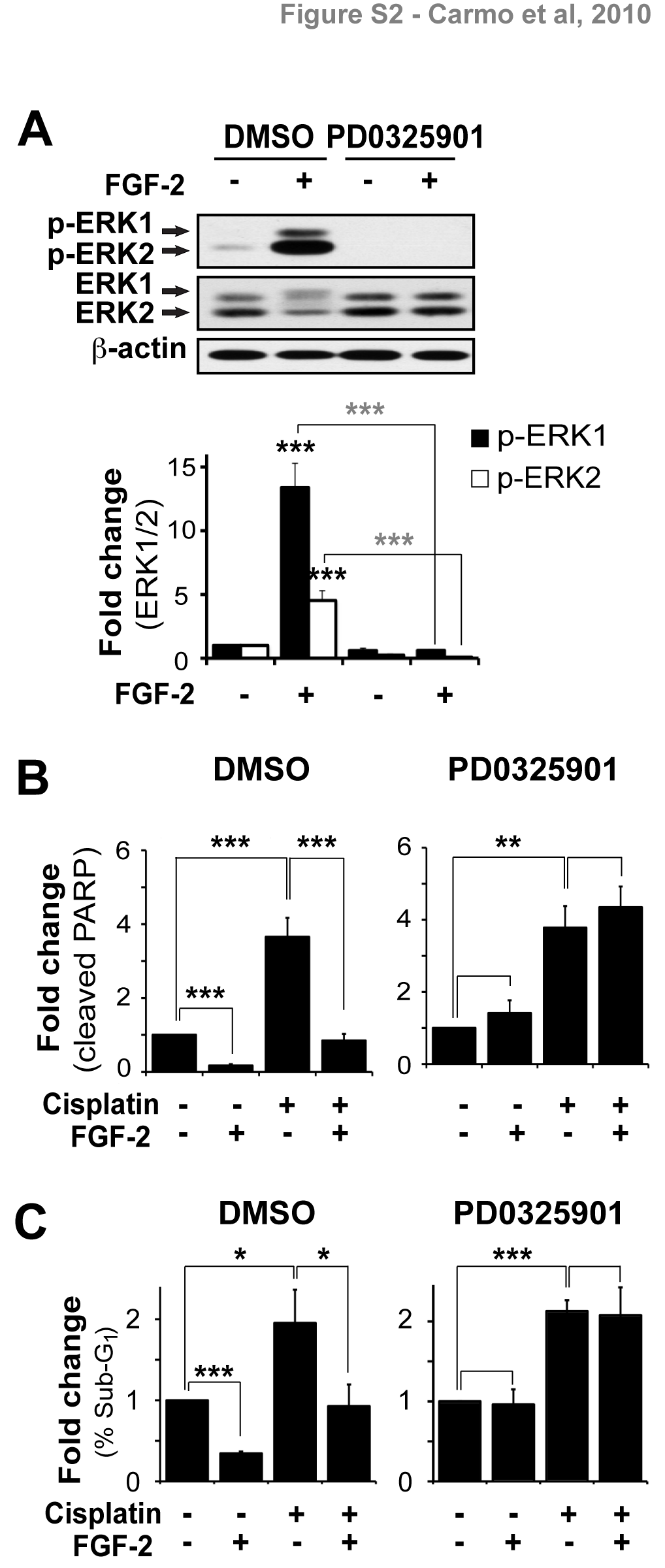

Supplement: Figure S2 — FGF-2-mediated drug resistance requires ERK1/2 phosphorylation. Cells were serum-starved (0.5% FCS) overnight, incubated in serum-free media for 1 hour and then pre-treated with 0.5 µM of PD0325901 or with drug vehicle [0.005% (v/v) DMSO] for 30 minutes. A. Cells were stimulated with 10 ng/ml of FGF-2 for 10 minutes. Proteins were extracted and separated on a 10% SDS-PAGE gel. Western blotting analysis was performed on total cell lysates using antibodies against pERK1/2-Thr202/185/Tyr204/187 and total ERK1/2. β-actin was used as a loading control. B. After 4 hours pre-treatment with FGF-2, cells were treated with cisplatin (60 µM) overnight, proteins were extracted from alive and dead cells, run on a 7.5% SDS-PAGE gel and analyzed by western blotting using a PARP antibody that recognizes cleaved PARP. C. Cells were treated as in (B.). After 18 h of cisplatin treatment, cells were harvested, permeabilized and the DNA stained. Apoptosis was assessed by flow cytometry using loss of DNA content as readout (% sub-G1 population). Mean±SEM from three independent experiments are shown in the graphs. Values are expressed as fold change over untreated controls (without PD0325901 and FGF-2). Statistical analysis was performed with Student's t-test (* – p<0.05, ** – p<0.01, *** – p<0.005 versus untreated control). [Grey asterisks in (A.): compare the indicated samples]. (TIF) [file pone.0019861.s002.tif]

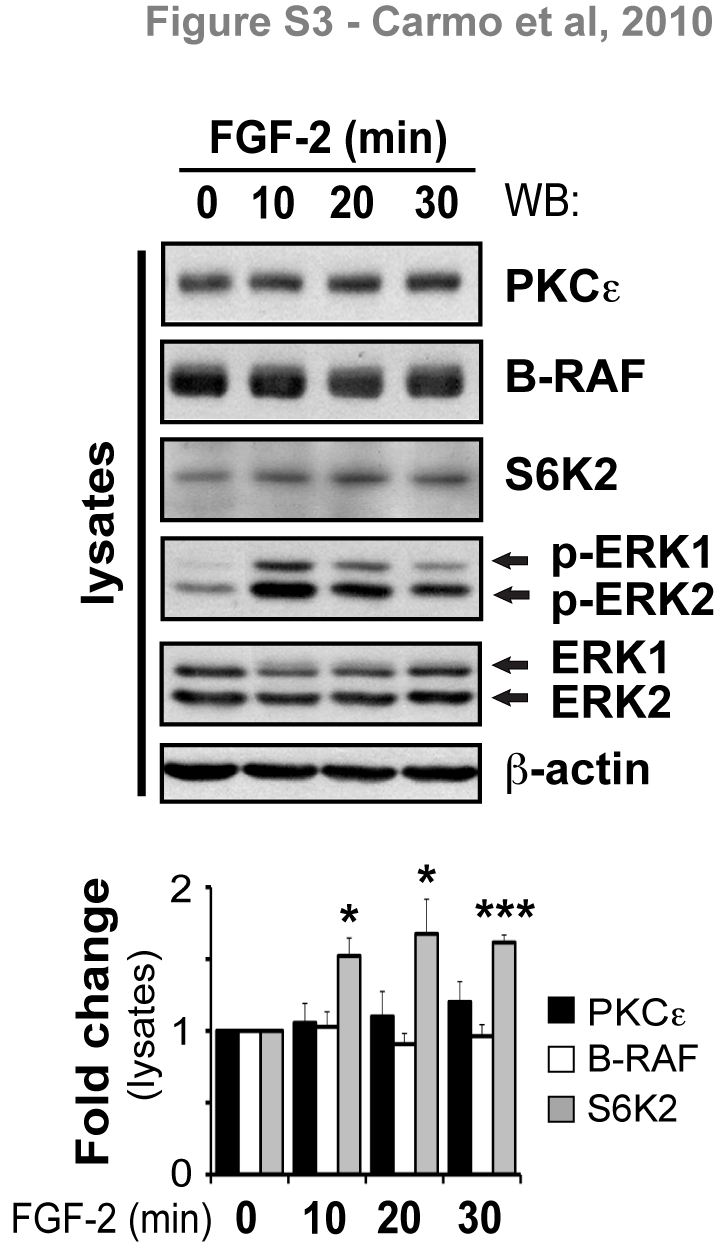

Supplement: Figure S3 — FGF-2 induced interactions between S6K2, PKCε and B-RAF. Whole cell lysates used to immunoprecipitate S6K2, PKCε and B-RAF were separated on a 7.5% SDS-PAGE gel and analyzed by western blot. Membranes were probed for PKCε, B-RAF, S6K2, pERK1/2-Thr202/185/Tyr204/187 and total ERK1/2. β-actin was used as a loading control. Representative western blots and mean±SEM of densitometric values from three independent experiments are shown in the graph. Values are expressed as fold change over untreated controls. Statistical analysis was performed with Student's t-test (* – p<0.05, *** – p<0.005 versus untreated controls). (TIF) [file pone.0019861.s003.tif]

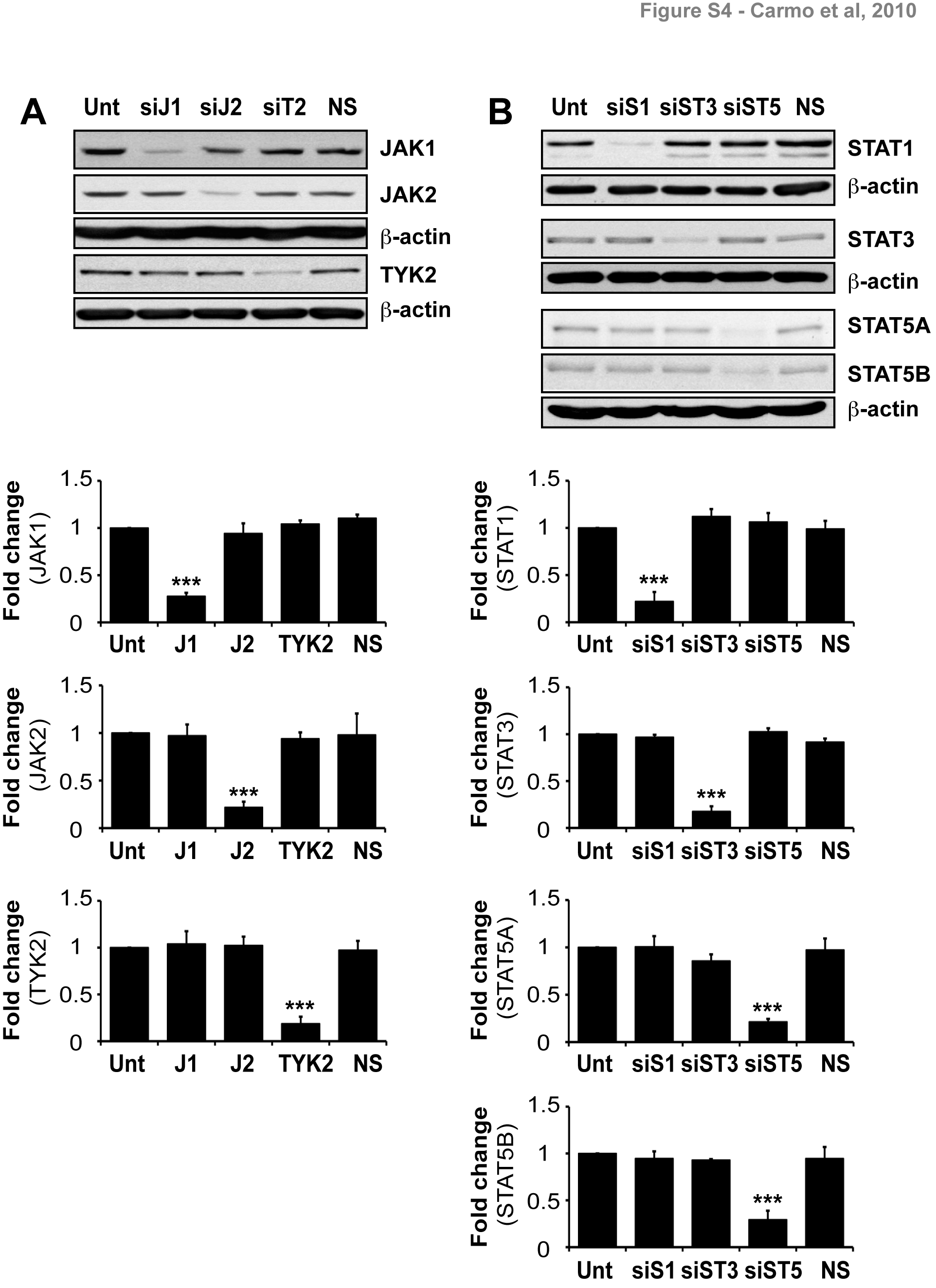

Supplement: Figure S4 — siRNA oligonucleotides can specifically knockdown TYK2, STAT1, STAT3 and STAT5A/B in U2OS cells. U2OS cells were transfected with 75 nM siRNA using DharmaFECT II. After 48 hours, proteins were extracted and separated on a 7.5% SDS-PAGE gel. Total cell lysates were western blotted as indicated. Cells were transfected with (A.) siTYK2, or (B.) with siRNA molecules targeting STAT1, STAT3 or STAT5A/B. Untransfected cells (Unt), or cells transfected with a non-specific siRNA (NS) were used as controls. Mean±SEM of densitometric values of three independent experiments are shown in graphs. Values are expressed as fold change relative to untransfected controls. Statistical analysis was performed with Student's t-test (*** – p<0.005 versus untransfected control). Unt – untransfected, NS – non-specific siRNA, T2 – TYK2, ST1 – STAT1, ST3 – STAT3, ST5 – STAT5A/B. (TIF) [file pone.0019861.s004.tif]

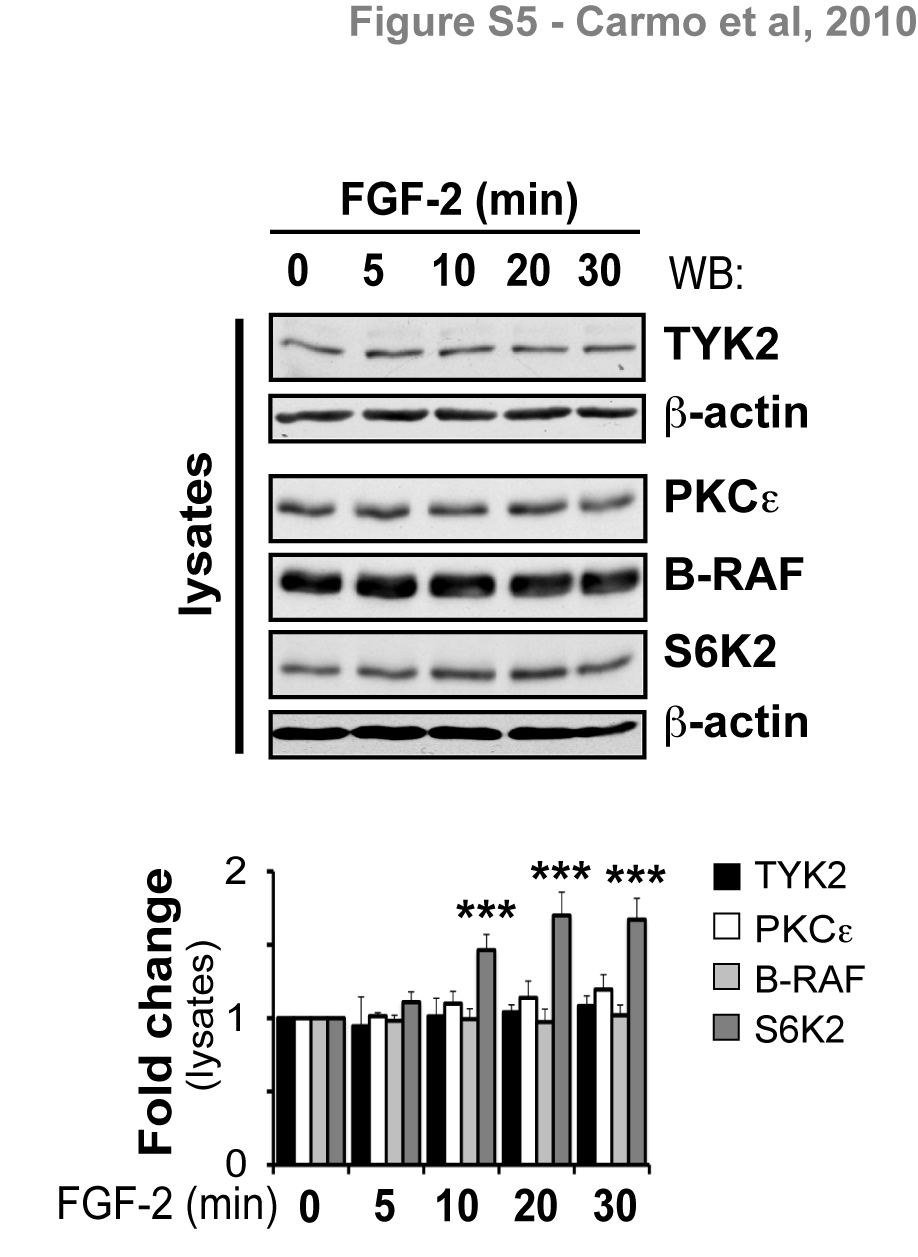

Supplement: Figure S5 — FGF-2 induced interactions between TYK2, PKCε and B-RAF. Whole cell lysates used to immunoprecipitate TYK2 were separated on a 7.5% SDS-PAGE gel and analyzed on a western blot. Membranes were probed for PKCε, B-RAF, S6K2 and TYK2. β-actin was used as a loading control. Representative western blots and mean±SEM of densitometric values from three independent experiments are shown in the graph. Values are expressed as fold change over untreated controls. Statistical analysis was performed with Student's t-test (*** – p<0.005 versus untreated controls). (TIF) [file pone.0019861.s005.tif]
